# Partial epithelial-mesenchymal transition in keloid scars: regulation of keloid keratinocyte gene expression by transforming growth factor-β1

**DOI:** 10.1186/s41038-016-0055-7

**Published:** 2016-08-23

**Authors:** Jennifer M. Hahn, Kevin L. McFarland, Kelly A. Combs, Dorothy M. Supp

**Affiliations:** 1 Research Department, Shriners Hospitals for Children – Cincinnati, Cincinnati, OH USA; 2 Department of Surgery, University of Cincinnati College of Medicine, Cincinnati, OH USA

**Keywords:** Keloid, Keratinocyte, Epithelial-mesenchymal transition, Wound healing, Scar, Fibrosis

## Abstract

**Background:**

Keloids are an extreme form of abnormal scarring that result from a pathological fibroproliferative wound healing process. The molecular mechanisms driving keloid pathology remain incompletely understood, hindering development of targeted, effective therapies. Recent studies in our laboratory demonstrated that keloid keratinocytes exhibit adhesion abnormalities and display a transcriptional signature reminiscent of cells undergoing epithelial-mesenchymal transition (EMT), suggesting a role for EMT in keloid pathology. In the current study, we further define the EMT-like phenotype of keloid scars and investigate regulation of EMT-related genes in keloid.

**Methods:**

Primary keratinocytes from keloid scar and normal skin were cultured in the presence or absence of transforming growth factor beta 1 (TGF-β1) +/− inhibitors of TGF-β1 and downstream signaling pathways. Gene expression was measured using quantitative polymerase chain reaction. Migration was analyzed using an in vitro wound healing assay. Proteins in keloid scar and normal skin sections were localized by immunohistochemistry. Statistical analyses utilized SigmaPlot (SyStat Software, San Jose, CA) or SAS^®^ (SAS Institute, Cary, NC).

**Results:**

In keloid and normal keratinocytes, TGF-β1 regulated expression of EMT-related genes, including hyaluronan synthase 2, vimentin, cadherin-11, wingless-type MMTV integration site family, member 5A, frizzled 7, ADAM metallopeptidase domain 19, and interleukin-6. Inhibition of canonical TGF-β1 signaling in keloid keratinocytes significantly inhibited expression of these genes, and TGF-β1 stimulation of normal keratinocytes increased their expression. The inhibition of the extracellular signal-regulated kinase 1/2 (ERK1/2) signaling pathway or the p38 mitogen-activated protein kinase pathway attenuated TGF-β1-induced expression of subsets of these genes. Migration of keloid keratinocytes, previously shown to be increased compared with normal keratinocytes, was significantly reduced by inhibition of TGF-β1 or ERK1/2 signaling. Biomarkers of EMT, including reduced E-cadherin and increased active β-catenin, were observed in keloid epidermis in vivo. However, evidence of basement membrane breakdown in keloid scar was not observed.

**Conclusions:**

The results suggest that keloid keratinocytes exist in an EMT-like metastable state, similar to activated keratinocytes in healing wounds. The EMT-like gene expression pattern of keloid keratinocytes is regulated by canonical and non-canonical TGF-β1 signaling pathways. Therefore, interventions targeting TGF-β1-regulated EMT-like gene expression in keloid keratinocytes may serve to suppress keloid scarring.

**Electronic supplementary material:**

The online version of this article (doi:10.1186/s41038-016-0055-7) contains supplementary material, which is available to authorized users.

## Background

Keloid scars are disfiguring fibrous growths that result from an abnormal wound healing process in susceptible individuals [1, 2]. Depending on their number, size, and location, keloids can negatively impact quality of life and disrupt activities of daily living [3]. Unlike hypertrophic scars, keloids tend to increase in size indefinitely, spreading beyond the original wound border although without metastasis to distant sites. Keloid scars are responsible for an estimated 427,000 physician visits per year in the USA; however, the total number of affected individuals may be greater because many people do not seek medical care [4]. The frequency of keloids is significantly higher in populations with darker skin pigmentation, such as African Americans and Hispanics [4–6]. By the middle of this century, the ethnic groups at greatest risk for keloid scarring will make up over half of the US population [7]. Therefore, keloids affect a large and increasing patient population. Keloids are a difficult clinical problem because they are refractory to treatment. Many therapies have low to moderate response rates or are associated with high rates of recurrence [1, 2]. High quality evidence is lacking for most current keloid treatments, despite their widespread use [8]. The underlying molecular pathology of keloid scarring remains incompletely understood, hindering development of more effective therapeutic interventions [1].

Keloids are characterized by abnormal and excessive deposition of extracellular matrix (ECM). Thus, fibroblasts have been the focus of the majority of keloid research to date. However, it is well recognized that keratinocytes are involved in regulation of fibroblast activity during wound healing, and numerous recent studies suggest that they have an important role in abnormal scar formation. We previously described the gene expression profile of keloid keratinocytes, which revealed aberrant expression of numerous genes involved in epithelial-mesenchymal transition (EMT), suggesting involvement of EMT in keloid pathology [9]. In EMT, epithelial cells lose their epithelial character, gain mesenchymal traits, and become less adhesive and more migratory [10, 11]. Three types of EMT have been described [12, 13]. Type I EMT occurs during embryonic development to enable cells to take on different fates during tissue morphogenesis and organ development. Type II EMT occurs in the context of tissue injury as part of the repair process, and is associated with inflammation. Type III EMT occurs during cancer progression, enabling epithelial-derived cancers to metastasize to distant sites. EMT is considered a transient process that can be reversed; for example, when metastatic cancer cells form a secondary tumor at a new location. Type II EMT ceases or reverses upon wound healing and resolution of inflammation [11]. However, in the presence of chronic inflammation, the repair process can be prolonged, resulting in excess deposition of ECM and formation of a fibrotic scar [14]. Thus, type II EMT has been implicated in tissue fibrosis [11, 15]. In fibrotic tissues, such as the kidney, it has been proposed that type II EMT contributes directly to fibrosis by causing epithelial cells to migrate locally through breaks in the basement membrane into the mesenchymal compartment, where they are reprogrammed to become ECM-producing fibroblasts [16]. In vitro treatment of epithelial cells with profibrotic cytokines can cause changes resembling EMT, but whether this actually occurs in vivo has been somewhat controversial as it is difficult to document [17].

Although the three types of EMT represent distinct and plastic physiological processes, they share a common set of phenotypic changes and are regulated by similar sets of signaling pathways and effector molecules [18]. Characteristic changes include alterations in cell surface markers, including a switch from the epithelial adhesion molecule E-cadherin (cadherin 1, type 1; CDH1) to *N*-cadherin (cadherin 2, type 1; CDH2) or OB-cadherin (cadherin-11, type 2; *CDH11*); changes in cytoskeletal proteins, including upregulation of the mesenchymal marker vimentin; increased expression of β-catenin, with decreased membrane localization and increased cytoplasmic and nuclear levels; and changes in transcription factors and basement membrane components [10, 19]. A “core” set of genes that are up- or downregulated during the EMT process has been identified, which includes genes involved in adhesion and migration; development, differentiation, and proliferation; angiogenesis and wound healing; and metabolism [20]. We found that many of these core EMT genes are mis-expressed in keloid keratinocytes, suggesting that EMT may have a central role in keloid pathology [9]. Compared with normal keratinocytes, keloid keratinocytes exhibit reduced expression of genes involved in cell-cell adhesion, altered expression of genes involved in epidermal development and differentiation, and increased expression of mesenchymal markers and biomarkers of EMT. In vitro, keloid keratinocytes display increased motility compared with normal keratinocytes. Keloid scars in vivo exhibit adhesion defects, including abnormal localization of desmosome components plakophilin 1 and junction plakoglobin (also known as *γ*-catenin), a core EMT gene that encodes a component of adherens junctions [9], and keloid scars were found to exhibit ultrastructural alterations of the basement membrane zone (BMZ) [21]. Recently, evidence of EMT in keloid scar tissue was reported, including enhanced expression of TGF-β1 and phosphorylated Smad3 [22]. However, unlike type III EMT in cancer, which is associated with basement membrane breakdown, keloid scars are not known to metastasize to distant sites. Further, if type II EMT is occurring in keloid scars, the migration of keratinocytes to the dermal compartment and their transition to mesenchymal cells has yet to be documented. Understanding the pathways that control the EMT-like gene expression profile of keloid keratinocytes, and better defining the EMT-like characteristics of keloid scars, may increase our understanding of the underlying pathology for development of improved, targeted therapies.

Transforming growth factor β1 (TGF-β1) is a multifunctional, profibrotic cytokine that is a major inducer of all three types of EMT [13, 23]. In canonical TGF-β1 signaling, binding of TGF-β1 to its receptors activates Smad2/3-dependent signaling; activated Smad2/3 complexes with Smad4 and translocates into the nucleus to regulate expression of TGF-β-responsive genes [12, 24]. During EMT, TGF-β1 signaling also occurs via non-canonical, Smad-independent pathways. For example, members of the mitogen-activated protein kinase (MAPK) family, such as ERK1/2 and p38 MAPKs, have been implicated in mediating many of the pathological activities of TGF-β1, such as tumor cell metastasis [24]. TGF-β1 has been shown to have a central role in keloid fibroblast pathology [25]. TGF-β1 is overexpressed in keloid fibroblasts [26] and contributes to abnormal extracellular matrix production in these cells [27]. TGF-β1 signaling via both canonical and non-canonical pathways has been implicated in abnormal gene expression in keloid fibroblasts [25, 28–30]. However, it is not clear whether TGF-β1 is involved in regulating abnormal gene expression in keloid keratinocytes. Treatment of normal epidermal keratinocytes with TGF-β1 in vitro has been shown to induce EMT biomarkers [31]. In mammary epithelial cells [32] and corneal keratocytes [33], TGF-β1 is a potent inducer of hyaluronan synthase 2 (*HAS2*), an enzyme involved in hyaluronic acid biosynthesis that mediates EMT in these cells. We previously reported that *HAS2* is significantly upregulated in keloid keratinocytes and contributes to their enhanced motility in vitro [9, 34]. In the HaCaT keratinocyte cell line, a line of spontaneously immortalized human epidermal keratinocytes, TGF-β1-induced EMT involved a set of genes that functions in cell-matrix adhesion and migration [35]. Hypothetically, TGF-β1 may regulate EMT-related abnormalities of keloid keratinocytes. The goal of the current study was to better understand the putative role of EMT in keloid scarring, and to determine the mechanisms that regulate EMT in keloid keratinocytes.

## Methods

### Human tissue samples: ethics and consent

Keloid scar and normal skin samples were obtained with approval of the University of Cincinnati Institutional Review Board (IRB), in accordance with the Declaration of Helsinki Principles, from patients at the Shriners Hospitals for Children - Cincinnati and the University of Cincinnati Medical Center (Table 1). Keloid scar samples were obtained with written informed consent from patients undergoing elective scar excision procedures. Written consent was obtained from parents or legal guardians of participants under the age of 18, with written assent obtained from pediatric patients age 14 or over, prior to sample collection. Patient information was anonymized, and samples were de-identified prior to analysis. Collection of de-identified normal skin samples from plastic surgery procedures was classified as “not human subjects research” by the University of Cincinnati IRB and was done without patient consent using discarded tissue. Strain numbers were used to enable de-identification and were assigned sequentially to all skin or scar samples collected by the laboratory, including those used for this study.

**Table 1:** Demographic data for donors of keloid and normal skin samples

Strain #	Age (years)	Gender	Race	Body site	Scar duration^a^
Keloid scar					
746K	15	Male	African American	Face	8 years^b^
795K	20	Female	African American	Neck	2.5 years^b^
797K	10	Male	White	Ear	2 years^b^
821K	12	Male	White	Abdomen	2 years
823K	17	Male	African American	Ear	1.5 years
843K	16	Male	White	Face	1 year
860K	9	Female	African American	Face	3 years
874K	22	Male	African American	Back	3 years^b^
Normal skin					
594	23	Male	White	Unknown	NA
737	16	Female	African American	Breast	NA
811	24	Female	African American	Breast	NA
815	17	Female	White	Breast	NA
877	17	Female	African American	Breast	NA
879	12	Female	White	Thigh	NA
880	15	Female	White	Breast	NA
886	17	Male	African American	Abdomen	NA

*NA* not applicable

^a^Approximate time in years since original injury or previous excision

^b^Recurrent scar

### Primary cell culture

Primary keratinocyte cultures were established as described elsewhere [9]. Briefly, tissue samples were cut into 2–3 mm strips and were incubated in Dispase II (Roche Applied Science, Indianapolis, IN) to separate dermis from epidermis. Epidermal strips were treated with 0.025 % trypsin (Sigma-Aldrich, St. Louis, MO), followed by neutralization with 10 % fetal bovine serum (Invitrogen/Thermo Fisher Scientific, Inc., Waltham, MA) and filtration through Falcon^®^ 70 μm cell strainers (Corning, Inc., Corning, NY) to release keratinocytes, which were inoculated into tissue culture flasks coated with collagen (Coating Matrix; Invitrogen/Thermo Fisher Scientific). Keratinocyte growth medium consisted of MCDB 153 with 0.06 mM calcium chloride [36], supplemented with 0.2 % bovine pituitary extract (Hammond Cell Tech, Windsor, CA), 1 ng/ml epidermal growth factor (EGF; PeproTech, Rocky Hill, NJ), 5 μg/ml insulin (Invitrogen/Thermo Fisher Scientific), 0.5 μg/ml hydrocortisone (Sigma-Aldrich), and 1× Penicillin–Streptomycin–Fungizone (Invitrogen/Thermo Fisher Scientific). Cells were subcultured when they reached 80–90 % confluence onto tissue culture flasks without collagen coating, using media as described above but with 0.2 mM calcium chloride. At passage 2, cells were harvested by trypsin treatment; 2 × 10^6^ cells per strain were used for isolation of RNA to compare gene expression in keloid and normal keratinocytes.

### TGF-β1 and inhibitor treatments

To analyze the effects of inhibition of TGF-β1 signaling in keloid keratinocytes, passage 3 cells of four donor strains (746K, 795K, 797K, 823K; see Table 1) were inoculated into six-well multiwell plates (Corning, Inc.) at 2000 cells/cm^2^ in keratinocyte growth medium (described above). After 48 h, the media was changed to keratinocyte growth medium without EGF, with or without (vehicle only) 1.0 μM SB525334 (Selleck Chemicals, Houston, TX), and cells were analyzed after 96 h. To analyze the effects of TGF-β1 on normal keratinocyte gene expression, passage 3 cells of four normal donor strains (594, 811, 880, and 886; see Table 1) were cultured in six-well plates as described above, with or without (vehicle only) 1.0 ng/ml recombinant human TGF-β1 (PeproTech). Media was refreshed after 48 h, and cells were lysed for RNA isolation after 96 h. To analyze inhibition of specific Smad-independent pathways downstream of TGF-β1 signaling, inhibitors were tested in combination with TGF-β1. To inhibit extracellular signal-regulated kinases 1 and 2 (ERK1/2) activation, cells were treated with 10 μM U0126 (Cell Signaling Technology, Danvers, MA), and for inhibition of p38 MAPK signaling, cells were treated with 10 μM SB203580 (EMD Millipore, Billerica, MA). At these concentrations, U0126 and SB203580 have been shown to inhibit ERK1/2 and p38 signaling, respectively, in normal human epidermal keratinocytes [37]. Keloid keratinocytes from four donor strains (746K, 795K, 797K, 823K) were inoculated at 25,000 cells/cm^2^ into six-well multiwell plates; 24 h later, cells were pre-incubated with inhibitors for 30 min prior to addition of 5 ng/ml TGF-β1. Cells were harvested for analysis after 24 h. All experiments were performed in duplicate for each strain analyzed.

### RNA isolation and gene expression analyses

Keratinocytes cultured in flasks (passage 2) were harvested by trypsin treatment and were pelleted prior to RNA isolation. Keratinocytes cultured in multiwell dishes (passage 3) were lysed without trypsin treatment. Total RNA was purified using RNeasy Mini Kits (Qiagen Inc., Valencia, CA). RNA samples were treated with DNase I (Qiagen, Inc.) prior to synthesis of cDNA using the SuperScript^®^ VILO cDNA Synthesis Kit (Invitrogen/Thermo Fisher Scientific). Quantitative PCR (qPCR) was performed using gene-specific primers (RT^2^ qPCR Primer Assays; Qiagen, Inc.), RT2 SYBR^®^ Green qPCR Mastermixes (Qiagen, Inc.) and the iCycler iQ system (BioRad, Hercules, CA). Technical triplicates were analyzed for each RNA sample, in addition to biological replicates. Expression levels were referenced to the glyceraldehyde 3-phosphate dehydrogenase (GAPDH) gene using the comparative 2^−∆∆Ct^ method [38]. For comparison of mean expression levels in normal and keloid keratinocytes (eight cell strains each), gene expression levels were normalized to mean expression in normal keratinocytes. For comparisons of mean expression levels in treated and untreated cells (four cell strains per experiment), expression levels were normalized to mean expression in untreated cells.

### In vitro wound healing assay

Keloid keratinocytes from four donors (strains 746K, 795K, 797K, and 823K) were cultured to confluence in six-well multiwell plates (Corning) coated with collagen (Coating Matrix; Invitrogen/Thermo Fisher Scientific). At time 0, media was changed to fresh growth media containing 10 μM U0126, 50 μM SB525334, or vehicle only (controls), and wounds were created by scratching the keratinocyte monolayer with a 200-μl pipet tip. Digital photographs were taken using phase-contrast microscopy at wounding and at 4, 8, 12, 16, and 24 h after wounding. Three wells per strain were analyzed, and three microscopic fields were photographed per well, for a total of nine fields per cell strain per time point. The bottoms of the wells were marked so that the same regions were photographed in each well at each time point. Image analysis was used to calculate the open area of each microscopic field (NIS-Elements AR3.1; Nikon, Melville, NY), and these values were used to calculate percent closure.

### Immunohistochemistry

Biopsies of keloid scar tissue or normal human skin were embedded in Shandon™ M1 Embedding Matrix (Invitrogen/Thermo Scientific, Waltham, MA). Cryosections were fixed by incubation in methanol followed by acetone at −20 °C. Immunohistochemistry was performed using standard techniques. Primary antibodies included anti-active-β-catenin mouse monoclonal antibody, clone 8E7 [39] (EMD Millipore, catalog #05-665), used at 1:100 dilution (16 h, 4 °C); anti-E-cadherin rabbit polyclonal antibody (Abgent Inc., San Diego, CA, catalog #AJ1249a), used at 1:500 dilution (1 h, room temperature); anti-vimentin rabbit polyclonal antibody [40] (Abgent, Inc., catalog # AJ1815a), used at 1:500 dilution (1 h at room temperature); anti-langerin mouse monoclonal antibody, clone 12D6 [41] (LifeSpan BioSciences, Inc., Seattle, WA, catalog #LS-C312086), used at 1:100 dilution (16 h, 4 °C); anti-tyrosinase-related protein 1 (TYRP1) mouse monoclonal antibody, clone Ta99 [42] (BioLegend, San Diego, CA, catalog #917801), used at 1:100 dilution (16 h, 4 °C); anti-laminin 5 mouse monoclonal antibody, clone P3H9-2 [43] (Abcam Inc., Cambridge, MA, catalog #ab78286), used at 1:500 dilution (1 h, room temperature); and anti-integrin alpha 6 rat monoclonal antibody, clone GoH3 [44] (BD Biosciences, San Jose, CA, catalog #555734), used at 1:100 dilution (1 h, room temperature). Labeled secondary antibodies (all from Invitrogen/Thermo Fisher Scientific) included Donkey anti-mouse IgG antibody, Alexa Fluor^®^ 488 conjugate (catalog #A21202); donkey anti-mouse IgG antibody, Alexa Fluor^®^ 594 conjugate (catalog #A21203); chicken anti-rabbit IgG antibody, Alexa Fluor^®^ 488 conjugate (catalog #A21441); donkey anti-rabbit IgG antibody, Alexa Fluor^®^ 594 conjugate (catalog #A21207); goat anti-rat IgG antibody, Alexa Fluor^®^ 594 conjugate (catalog #A21213); and donkey anti-rabbit IgG antibody, Alexa Fluor^®^ 350 conjugate (catalog #A10039). Secondary antibodies were diluted 1:400 and incubated with sections for 1 h at room temperature. Coverslips were mounted on slides using Vectashield^®^ Mounting Media (Vector Laboratories, Burlingame, CA), with or without 4,6-diamidino-2-phenylindole (DAPI) for counterstaining of cell nuclei. Sections were viewed with an Eclipse 90i microscope and photographed with a DS-Ri1 Digital Microscope Camera (Nikon Instruments Inc., Melville, NY).

### Statistical analyses

For all experiments, keratinocytes from different donors were cultured and analyzed independently; at no point were cells of different donors combined in vitro. Quantitative results are expressed as the mean (± standard deviation) of values for individual cell strains. Statistical analyses were performed using SigmaPlot for Windows Version 11.0 (SyStat Software, Inc., San Jose, CA) or SAS® Statistical Analysis Software (SAS Institute, Cary, NC). Differences were considered statistically significant at *p <* 0.05. Pairwise comparisons of numeric values were analyzed by *t* test. Pairwise comparisons of categorical data were analyzed using chi-square test. Comparisons involving more than two values were done using one-way analysis of variance (ANOVA) followed by multiple pairwise comparisons using Tukey’s test. For the wound healing assay, data were analyzed using a two-way analysis of variance with one repeated factor using the MIXED procedure in SAS, and multiple comparisons were done using the least squares means procedure following statistical significance of the main effects.

## Results

### Cell donor characteristics

Primary keratinocytes were cultured from eight keloid scars and eight normal full thickness skin samples collected from 16 different individuals. Normal skin samples were not collected from keloid scar patients for ethical reasons, as biopsies of the non-lesional skin would result in a high probability of new keloid scar formation in this pediatric to young adult patient population. Tissue samples were from White or African American patients of both genders, and from a variety of body sites (Table 1). Keloid scar samples were from patients ranging in age from 9 to 22 years (mean 15.13 ± 4.61 years), and normal samples were from patients ranging in age from 12 to 24 years (mean 17.63 ± 4.00 years). Four of the keloids were recurrent scars that returned following previous surgical excision. Differences in age, gender, and race between the keloid scar and normal skin donors were not statistically significant.

### EMT-like gene expression in keratinocytes cultured from keloid scars

**Fig. 1:**
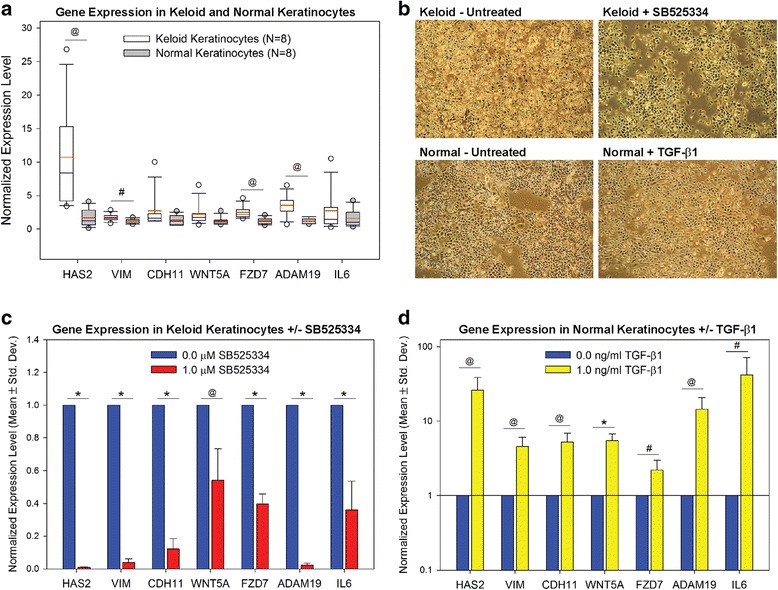
Altered EMT-related gene expression in keloid keratinocytes is regulated by TGF-β1 signaling. (**a**, **c**, **d**) Gene expression was analyzed by qPCR. Statistically significant differences are indicated by symbols: *p < 0.001; ^@^ *p <* 0.01; ^#^ *p <* 0.05. **a** A box plot was used to illustrate the variability in gene expression, particularly among keloid scar keratinocytes. *White bars* represent expression levels in keloid keratinocytes, and *gray bars* represent expression levels in normal keratinocytes. For each box, the box limits define the 75th percentile (*upper*) and 25th percentile (*lower*); the *black bar* within the box indicates the median; the *red bar* within box indicates the mean; the *whiskers* represent the 90th percentile (*upper*) and 10th percentile (*lower*); and *circles* represent outliers. **b** Experimental manipulation of TGF-β1 signaling in keloid and normal keratinocytes alters colony appearance in vitro. Shown are representative images of keloid keratinocytes cultured in the absence (*upper left*) or presence (*upper right*) of SB525334 to inhibit TGF-β1 signaling, and normal keratinocytes cultured in the absence (*lower left*) or presence (*lower right*) of TGF-β1. **c** Inhibition of TGF-β1 signaling in keloid keratinocytes with SB525334 normalizes the EMT-like gene expression pattern. **d** Stimulation of normal keratinocytes with TGF-β1 induces expression of genes involved in EMT

Expression levels for seven genes implicated in EMT were quantified in normal and keloid keratinocytes: hyaluronan synthase 2 (*HAS2*); vimentin (*VIM*); cadherin-11 (*CDH11*); wingless-type MMTV integration site family, member 5A (*WNT5A*); frizzled 7 (*FZD7*); ADAM metallopeptidase domain 19 (*ADAM19*); and interleukin-6 (*IL6*). These genes were selected for analysis because they were identified in previous expression profiling studies to be elevated in keloid keratinocytes compared with normal keratinocytes [9], and they represent different types of proteins previously shown in various contexts to be involved in EMT. *HAS2*, *VIM*, *CDH11*, and *WNT5A* are considered core EMT genes [20]. *HAS2*, among the most highly upregulated genes in keloid vs. normal keratinocytes, is one of three HAS genes involved in synthesis of hyaluronic acid. *HAS2* overexpression was sufficient to induce EMT in normal epithelial cells [45], and was demonstrated to mediate TGF-β1-induced EMT in several cancers [32, 46, 47]. *VIM*, an intermediate filament gene, and *CDH11*, which encodes a component of adherens junctions, are both considered markers of mesenchymal cells that are increased during EMT [10, 19, 20, 48]. *WNT5A*, a secreted signaling protein involved in canonical and non-canonical WNT signaling, and *FZD7*, a receptor for *WNT5A* and other WNT proteins, are both overexpressed in numerous cancers where they contribute to the EMT phenotype [49, 50]. *ADAM19* is involved in ectodomain shedding of cadherins, a proteolytic processing event that contributes to reduced cadherin protein levels during EMT [51, 52]. *ADAM19* is overexpressed in renal fibrosis where it is regulated by canonical TGF-β1 signaling [53]. *IL6* is a proinflammatory cytokine that induces expression of transcription factors involved in EMT, increases *VIM* expression, and enhances cell motility [54, 55]. The mean expression levels for these seven genes were higher in keloid keratinocytes than in normal keratinocytes (Fig. 1a). However, the differences were only statistically significant for *HAS2*, *VIM*, *FZD7*, and *ADAM19*. This is likely due to the high degree of variability in expression levels, particularly among keloid keratinocytes isolated from different donors.

### Regulation of EMT-like gene expression in keloid keratinocytes by canonical TGF-β1 signaling

TGF-β1 has been shown to induce EMT via canonical pathways, involving Smad signaling, and non-canonical, Smad-independent pathways [23]. To determine if elevated expression of EMT-related genes in keloid keratinocytes is regulated by canonical TGF-β1 signaling, cells were cultured for 4 days in the presence of SB525334, a potent and selective inhibitor of the TGFβ receptor I (activin receptor-like kinase; ALK5). SB525334 inhibits ALK5 kinase activity, and thereby inhibits TGF-β1-induced phosphorylation and nuclear translocation of Smad2 and Smad3 [56]. Treatment of keloid keratinocytes with SB525334 resulted in changes in morphology that suggest alteration of adhesive properties (Fig. 1b). Untreated keloid keratinocytes form looser colonies compared with normal keratinocytes, and appear more refractile under phase-contrast microscopy, indicating a more rounded shape while growing on plastic. Inhibition of TGF-β1 signaling by SB525334 treatment resulted in more tightly associated colonies, and flatter, less refractile cells, suggesting increased adhesive properties (Fig. 1b). Analysis of Smad3 phosphorylation in these cells (Additional File 1) is shown in Additional file 2: Figure S1. Expression levels in keloid keratinocytes for EMT-related genes *HAS2*, *VIM*, *CDH11*, *WNT5A*, *FZD7*, *ADAM19*, and *IL6* were significantly reduced by inhibition of TGF-β1 signaling with SB525334 (Fig. 1c).

To determine whether TGF-β1 regulates expression of the selected EMT-related genes in non-keloid cells, normal epidermal keratinocytes were cultured for 4 days with 1.0 ng/ml recombinant human TGF-β1. Compared with untreated normal keratinocytes, which tend to form tightly associated colonies in vitro, TGF-β1 treatment of normal keratinocytes resulted in looser colonies with greater separation among cells, a greater number of elongated cells, and an increased number of refractile cells, suggesting reduced adhesive properties, similar to keloid keratinocytes (Fig. 1b). Expression levels for all seven EMT-related genes were significantly increased in normal keratinocytes by TGF-β1 treatment (Fig. 1d).

### Involvement of non-canonical TGF-β1 signaling pathways in EMT-like keloid keratinocyte gene expression

**Fig. 2:**
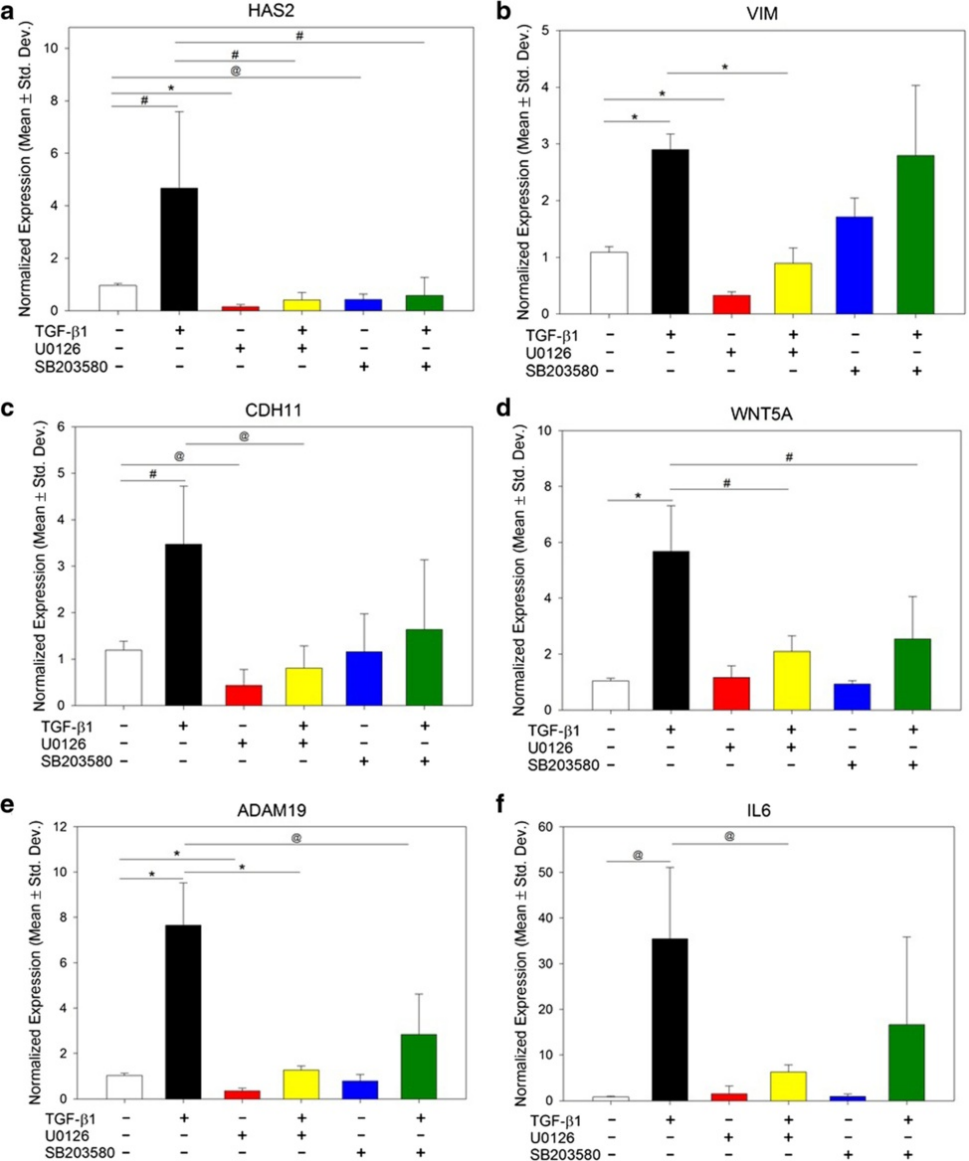
Inhibition of ERK1/2 and p38 MAPK signaling in keloid keratinocytes alters TGF-β1-induced gene expression. Keloid keratinocytes (four donor strains) were pre-incubated with inhibitors of ERK1/2 signaling (U0126) or p38 MAPK signaling (SB203580), prior to treatment with TGF-β1. Gene expression levels, quantified by qPCR, are shown for *HAS2* (**a**), *VIM* (**b**), *CDH11* (**c**), *WNT5A* (**d**), *ADAM19* (**e**), and *IL6* (**f**). *White bars*, no treatment; *black bars*, +TGF-β1; *red bars*, +U0126; *yellow bars*, +U0126 + TGF-β1; *blue bars*, +SB203580; *green bars*, +SB203580 + TGF-β1. Statistically significant differences are indicated by symbols: *p < 0.001; ^@^ *p <* 0.01; ^#^ *p <* 0.05

In addition to Smad-dependent signaling, TGF-β1 induces EMT through parallel, Smad-independent pathways, including ERK1/2 and p38 [23, 24]. To begin to investigate the role of non-Smad/MAP kinase pathways in TGF-β1-induced EMT gene expression in keloid keratinocytes, cells were pretreated with an inhibitor of ERK1/2 signaling, U0126, prior to stimulation with TGF-β1. For six of the seven genes tested—*HAS2*, *VIM*, *CDH11*, *WNT5A*, *ADAM19*, and *IL6*—TGF-β1 stimulation significantly increased expression in keloid keratinocytes, as was observed in normal keratinocytes, and this increase was significantly attenuated by inhibition of the ERK1/2 pathway with U0126 (Fig. 2). For *HAS2*, *CDH11*, *ADAM19*, and *VIM*, ERK1/2 inhibition also significantly inhibited basal transcription levels, in the absence of exogenous TGF-β1 (Fig. 2). Mean expression levels for *FZD7* were not significantly altered by either TGF-β1 stimulation or U0126 treatment.

Previous studies demonstrated that treatment of normal epidermal keratinocytes with the p38 inhibitor SB203580 attenuated the EMT-like phenotype induced by TGF-β1 [57]. To determine if p38 is involved in TGF-β1-regulated EMT-like gene expression in keloid keratinocytes, cells were cultured with or without TGF-β1 stimulation in the absence or presence of SB203580. Pretreatment of keloid keratinocytes with SB203580 significantly attenuated TGF-β1-induced expression of *HAS2*, *WNT5A*, and *ADAM19*, and basal expression of *HAS2* was significantly reduced by SB203580 treatment (Fig. 2). These results suggest that p38 contributes to regulation of a subset of EMT-related genes.

### Inhibition of TGF-β1 signaling impairs migration of keloid keratinocytes

**Fig. 3:**
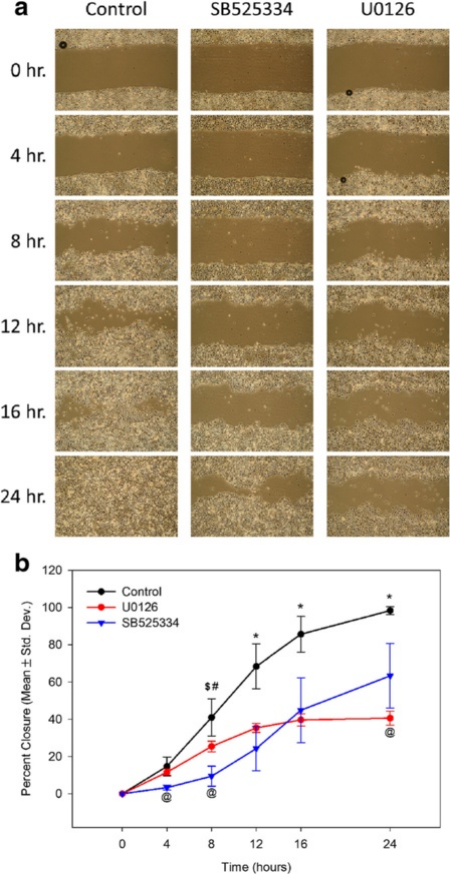
Inhibition of TGF-β1 or ERK1/2 signaling inhibits migration of keloid keratinocytes. **a** An in vitro wound healing assay was performed to measure the effects of inhibitors of TGF-β1 signaling (SB525334) or ERK1/2 signaling (U0126) on migration of keloid keratinocytes (*N* = 4 donor strains). Representative images of wound healing assay are shown. **b** Quantitative analysis of the in vitro wound healing assay. Mean values for four donor strains (+ standard deviations) are plotted vs. time (hours). Statistically significant differences at specific time points are indicated by symbols: *p < 0.001 control vs. both other groups; ^$^ *p <* 0.001 control vs. SB525334; ^#^ *p <* 0.02 control vs. U0126; ^@^ *p <* 0.05 SB525334 vs. U0126

Previous studies demonstrated that keloid keratinocytes display increased migration rates compared with normal keratinocytes [9]. To investigate the role of TGF-β1/Smad and ERK1/2 signaling in keloid keratinocyte migration, an in vitro wound healing assay was performed. Keloid keratinocytes were treated at the time of wounding with either SB525334 or U0126. Both treatments inhibited wound closure, with significant delays compared with controls starting at 8 h after wounding (Fig. 3). Interestingly, the kinetics of closure was slightly different between the two treatment groups. Significant differences between SB525334-treated and U0126-treated keratinocytes were observed at 4 and 8 h after wounding, with U0126-treated cells exhibiting higher migration rates during this early period. No significant differences were observed at 12 and 16 h after wounding, but by 24 h, migration of U0126-treated cells reached a plateau, and wound closure at this time point was significantly lower than in SB525334-treated cells (Fig. 3).

### Biomarkers of EMT in keloid scar tissue

**Fig. 4:**
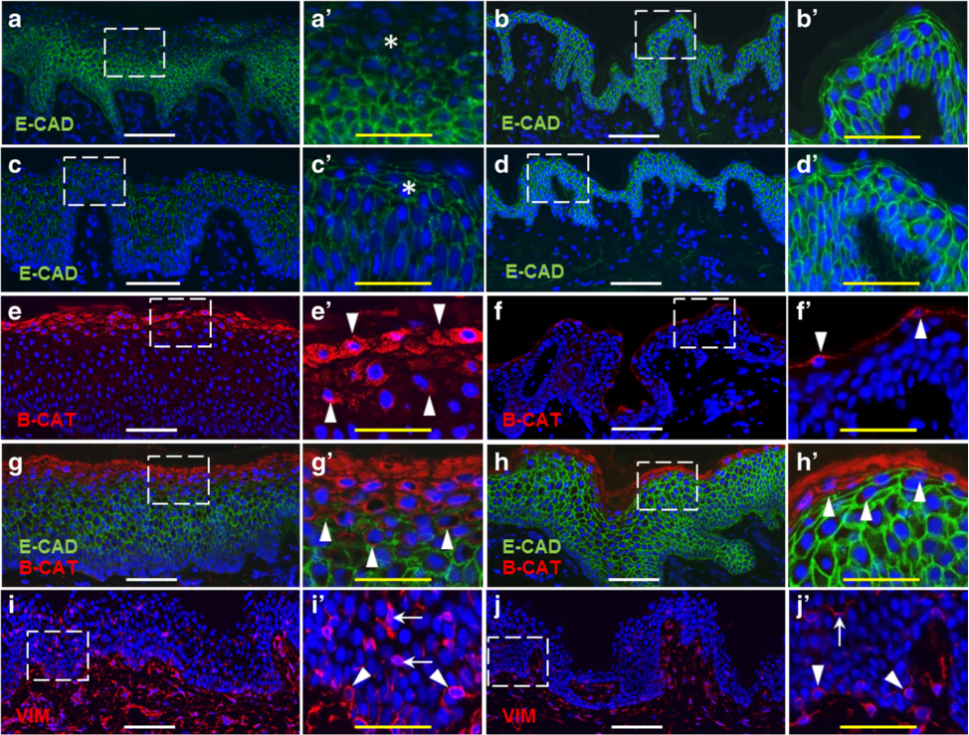
Analysis of EMT biomarkers in normal human skin and keloid scar tissue. Immunohistochemistry was performed using antibodies against E-cadherin (*green*; **a-d**, **g-h**), active β-catenin (*red*; **e**-**h**), and vimentin (*red*; **i**-**j**). Nuclei were counterstained with DAPI (*blue*) in all sections. Different keloid scar sections are shown in *left panels* (**a**, **c**, **e**, **g**, and **i**). Normal skin sections are shown in right panels (**b**, **d**, **f**, **h**, and **j**). Panels **a’**–**j’** are threefold magnified images of the boxed regions shown in **a**–**j**. (**a**–**d**) E-cadherin (*green*) is reduced in keloid scar epidermis (**a**, **a’**, **c**, **c’**) compared with normal epidermis (**b**, **b’**, **d**, **d’**). Variable staining intensity is observed among keloids from different individuals, with relatively low levels in the upper dermal layers (*asterisks* in **a’** and **c’**) compared with normal epidermis. (**e**–**h**) Regions of active β-catenin expression (*red*; indicated by *arrowheads* in **e’**–**h’**) are increased in keloid epidermis (**e**, **g**) compared with normal epidermis (**f**, **h**). **g**, **h** E-cadherin and β-catenin show complementary expression patterns in keloid epidermis. **i**, **j** Vimentin (*red*) is increased in keloid scar (**i**-**i’**) compared with normal skin (**j**-**j’**). Epidermal vimentin staining is observed in cells of the basal layer (*arrowheads*) and within the upper epidermal layers (*arrows*). *White scale bars* (**a**–**j**), 100 μm; *yellow scale bars* (**a’**–**j’**), 50 μm. Representative images are shown

Immunohistochemistry was used to localize biomarkers of EMT in keloid scar tissue. A previous study reported features of EMT in keloid scar epidermis, including increased levels of TGF-β1 and phosphorylated Smad3 compared to normal epidermis, and decreased E-cadherin levels [22]. Decreased expression of E-cadherin, a component of epithelial adherens junctions, is considered a hallmark of EMT [10]. We did not observe a statistically significant difference in expression of the CDH1 gene, which encodes E-cadherin, between normal and keloid keratinocytes (data not shown). However, we observed reductions in E-cadherin protein levels in keloid scars compared with normal human skin biopsies (Fig. 4a–d). The levels were variable among different scars samples collected from different patients. Figure 4a–d illustrates the variable expression levels observed in two different scars compared with two normal skin samples. Although there was a trend towards reduced E-cadherin levels in larger, more rapidly expanding keloids, there were insufficient numbers of patients available to establish a statistically significant correlation between keloid severity and E-cadherin expression (data not shown). Interestingly, membrane localization of E-cadherin showed a greater reduction in the upper epidermal layers, whereas localization in normal epidermis was similar at all epidermal levels (Fig. 4A’–D’).

Another characteristic feature of EMT, increased levels of cytoplasmic and nuclear β-catenin, was assessed in keloid scar tissue. At the transcriptional level, we did not detect significant differences in β-catenin expression between keloid and normal keratinocytes, although β-catenin expression was induced by TGF-β1 treatment of keloid keratinocytes (Additional file 3: Figure S2). To examine protein localization, immunohistochemistry was performed using an antibody to the active form of β-catenin, which is dephosphorylated on Ser37 or Thr41 [58]. Active β-catenin was detected in keratinocytes in the uppermost nucleated layers of the epidermis in both keloid scar and normal epidermis (Fig. 4e–h). However, a broader band of expression was observed in keloid epidermis, with three to five cell layers showing positive staining for active β-catenin, compared with only one to two cell layers in normal skin. The epidermal regions with the highest levels of active β-catenin staining in keloid scars corresponded to the regions with the lowest E-cadherin levels (Fig. 4g).

As described above, expression levels of the mesenchymal cell marker *VIM* were significantly higher in keloid vs. normal keratinocytes in vitro. In keloid scar tissue, levels of vimentin were higher in keloid epidermis compared with normal epidermis (Fig. 4i, j), as was previously reported by others [22]. In addition to positive intra-epidermal vimentin staining, we observed vimentin-positive cells in the basal epidermal layer (Fig. 4l). Interestingly, similarly localized vimentin-positive cells were also observed in normal epidermis (Fig. 4j).

### Analysis of basement membrane integrity in keloid scars

Basement membrane breakdown is a critical step in type I EMT during embryogenesis [59] and in adults type III EMT in cancer during the progression to malignancy [60, 61]. Transition of epithelial cells to fibroblasts that transverse the basement membrane and migrate into the mesenchyme is a feature of type II EMT that promotes pathological fibrosis [61]. A previous report examined vimentin expression in hypertrophic scars and found vimentin-positive cells in proximity to the BMZ [62]. In that study, partial breakdown of the basement membrane was observed in proximity to epidermal cells expressing mesenchymal markers, suggesting EMT accompanied by migration of cells from the epidermis into the dermis [62]. Ultrastructural studies of keloid scars previously showed abnormalities of the BMZ, with decreased desmosome length and reduced hemidesmosome density in keloid scars compared with normal skin [21]. The identification of increased vimentin expression in keloid scars and localization of vimentin-positive cells in the basal epidermal layer prompted us to examine localization of basement membrane proteins in keloids to investigate whether the locally invasive behavior of keloid scars may involve EMT-related breakdown of the basement membrane.

**Fig. 5:**
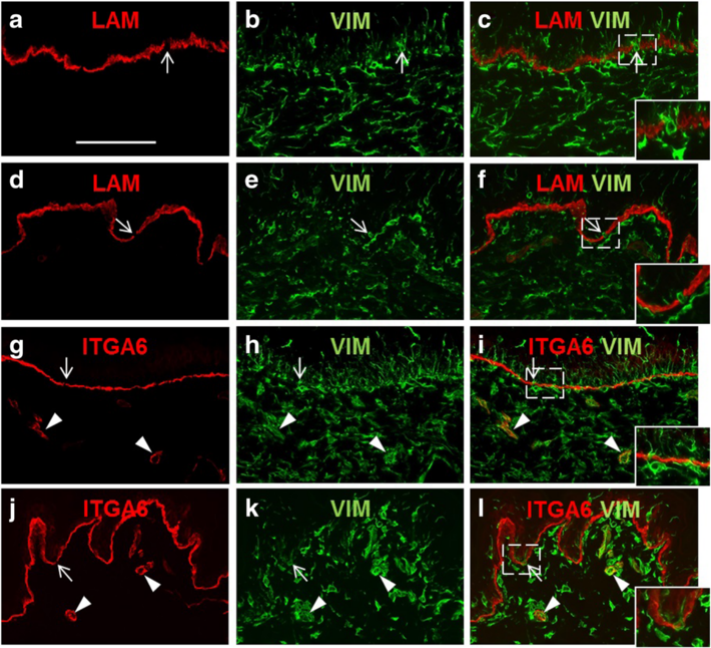
Localization of basement membrane components and vimentin in keloid scar tissue and normal skin. (**a**–**f**) Immunohistochemical localization of laminin-332 (LAM; *red*) and vimentin (VIM; *green*) in keloid scar (**a**–**c**) and normal skin (**d**–**f**). **g**–**l** Localization of integrin α6 (ITGA6; *red*) and vimentin (VIM; *green*) in keloid scar (**g**–**i**) and normal skin (**j**–**l**). A single section is shown in each row. Panels in the *right column* (**c**, **f**, **i**, **l**) show merged images; *inset* images are twofold magnifications of boxed areas. *Arrows* indicate vimentin-positive cells in regions with apparent gaps in basement membrane staining for laminin-332 or integrin α6. *Arrowheads* (**g**–**l**) indicate blood vessels displaying colocalization of integrin α 6 and vimentin in keloid scar (**g**–**i**) and normal skin (**j**–**l**). All panels are shown at the same magnification; *scale bar* in panel **a**, 100 μm

Laminin-332 (formerly known as laminin-5) secreted by keratinocytes is a major component of the basement membrane [63]. Laminin-332 interacts with other ECM components, such as collagen type IV and collagen type VII, and it complexes with integrin α6 to form a major component of hemidesmosomes [63]. Discontinuities of laminin-332 and integrin α6 BMZ staining were identified in keloid epidermis, and the observed breaks in basement membrane staining were associated with vimentin-positive cells in the basal epidermal layer (Fig. 5a–c, g–i). Similar vimentin-expressing cells were observed near the BMZ of normal epidermis, adjacent to regions with apparent gaps in laminin-332 or integrin α6 expression (Fig. 5d–f, j–l). In both keloid scars and normal skin, positive staining for integrin α6, which was previously reported to be expressed in endothelial cells [64], was identified in blood vessels and was co-localized with vimentin (Fig. 5g–i). Localization of collagen IV, a type of collagen expressed in the BMZ of the skin, showed linear expression at the dermal-epidermal junctions of both keloid scars and normal skin, with no discernable differences or breaks in staining (data not shown).

**Fig. 6:**
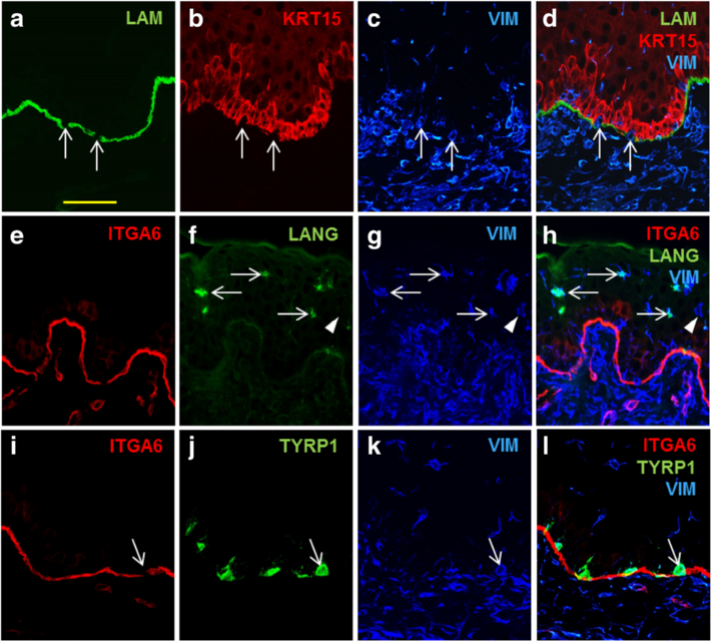
Characterization of vimentin-positive cells in keloid epidermis. Immunohistochemistry was performed to localize vimentin and markers for basement membrane, basal keratinocytes, Langerhans cells, and melanocytes in keloid scar tissue. (**a**–**d**) Localization of laminin-332 (LAM; *green*, **a**), keratin 15 (KRT15; *red*, **b**), and vimentin (VIM; *blue*, **c**), and merged image (**d**). *Arrows* indicate cells at gaps in basement membrane that are negative for keratin 15 and positive for vimentin. (**e**–**h**) Localization in keloid epidermis of integrin α6 (ITGA6; *red*, **e**), langerin (LANG; *green*, **f**), and vimentin (VIM; *blue*, **g**), and merged image showing colocalization (**h**). *Arrows* indicate examples of cells positive for both langerin and vimentin; *arrowhead* indicates vimentin-positive, langerin-negative epidermal cell. (**i**–**l**) Localization in keloid scar of integrin α6 (ITGA6; *red*, **i**), tyrosinase-related protein 1 (TYRP1; *green*, **j**), vimentin (*blue*, **k**), and merged image showing colocalization (**l**). A single section is shown in each row. *Scale bar* in **a** is same for all sections, 50 μm

To better characterize the vimentin-positive cells in keloid epidermis, immunohistochemistry was performed using markers for different types of epidermal cells. Vimentin-positive cells adjacent to breaks in laminin-332 staining at the basement membrane were negative for keratin 15, a basal keratinocyte marker, suggesting they are not keratinocytes (Fig. 6a–d), an observation consistent with keratinocytes transitioning to mesenchymal cells. However, closer examination of the intra-epidermal vimentin-positive cells revealed a dendritic morphology, suggesting that at least some of these cells may be Langerhans cells, which were previously reported to express vimentin [65]. Colocalization of the Langerhans cell marker, langerin, with some vimentin-positive cells in keloid scar epidermis was observed, suggesting that many of the vimentin-positive epidermal cells in keloid scars are Langerhans cells (Fig. 6e–h). Further, many of the vimentin-positive cells located near the BMZ in keloid scars, adjacent to gaps in integrin α6 staining, were positive for the melanocyte-specific protein tyrosinase-related protein 1 (TYRP1), indicating these cells are melanocytes (Fig. 6i–l).

## Discussion

A limitation of many current treatments for keloid scarring, such as intralesional corticosteroid injection, surgical excision, and 5-fluorouracil, is that they treat the symptom, not the disease. They do not correct the underlying pathology, which is not yet fully understood, and thus are associated with high rates of recurrence [66]. Many of the currently available therapies target fibroblast activity. Hypothetically, these interventions may fail to provide long-term suppression of keloid fibrosis because other cell types, in addition to fibroblasts, are driving keloid pathology.

Keratinocytes are critical regulators of fibroblast activity during wound healing, and recent studies suggest that the epidermis can play an active role in dermal fibrosis [67–70]. Defects in epidermal integrity can promote fibrosis indirectly by increasing inflammation, which leads to profibrotic stimulation of fibroblasts, or directly by secretion of paracrine factors that regulate fibroblast proliferation and matrix production. Additionally, it has been proposed that keratinocytes can directly contribute to fibrosis via the process of EMT [11]. Previous expression profiling studies from our laboratory strongly suggested a role for EMT in keloid pathology [9]. Here, we extend those studies to show that multiple genes with varying functions during EMT are upregulated in keloid keratinocytes, and that expression of these genes in both keloid and normal cells is regulated by TGF-β1. Further, the induction of EMT-related genes in keloid keratinocytes is at least partially mediated by ERK1/2 signaling, as inhibition of the ERK1/2 pathway attenuated the TGF-β1-stimulated increase in gene expression. In a subset of these genes, induction of expression by TGF-β1 was also partially mediated by p38.

TGF-β1-regulated expression of *HAS2* [32], *VIM* [71], *CDH11* [48], *WNT5A* [72], *IL6* [73], and *ADAM19* [74] has been previously implicated in EMT and fibrosis. Although *FZD7* has been previously implicated in EMT [50], the regulation of *FZD7* by TGF-β1 signaling has not been previously described. In the current study, *FZD7* expression was induced in normal keratinocytes by TGF-β1 treatment and was downregulated in keloid keratinocytes by inhibition of TGF-β1 signaling. These results implicate TGF-β1 in regulation of *FZD7* in keloid and normal keratinocytes. However, in experiments performed to analyze the effects of ERK1/2 and p38 inhibitors on TGF-β1-induced gene expression, significant differences in *FZD7* expression were not observed. This apparent discrepancy may be due to differences in the treatment conditions (inoculation density, culture time, and/or TGF-β1 concentration) used for these experiments. Alternatively, exogenous TGF-β1 may be insufficient to upregulate *FZD7* expression in keloid keratinocytes, which is already significantly elevated compared with normal keratinocytes. Further, it is possible that non-canonical ERK1/2 and p38 pathways are not involved in TGF-β1-regulated expression of this gene in keloid keratinocytes.

In addition to inducing upregulation of EMT-related genes, treatment of normal epidermal keratinocytes with TGF-β1 changed their in vitro appearance, resulting in less tightly adhered colonies. The phenotype of keloid keratinocytes in vitro, which is characterized by loose cell-cell and cell-substrate attachment and increased motility, was partially normalized by inhibition of TGF-β1 signaling. Inhibition of canonical TGF-β1 signaling by SB525334 treatment significantly reduced keloid keratinocyte migration rates in vitro. Inhibition of ERK1/2 signaling also inhibited migration of keloid keratinocytes, but with different kinetics than inhibition of canonical TGF-β1 signaling. Taken together, the results suggest that the TGF-β1/Smad2/3 and ERK1/2 signaling pathways regulate overlapping but partially distinct sets of genes to regulate migration and adhesion in keloid keratinocytes. Hypothetically, simultaneous inhibition of both canonical and non-canonical pathways downstream of TGF-β1 may result in additive effects, as observed in other fibrotic disorders [75], although this was not addressed in the current study.

Our previous studies identified adhesion abnormalities in keloid scar epidermis in vivo, including reduced expression of junction plakoglobin and plakophilin 1 [9]. Recently, Yan et al. reported additional evidence of EMT in keloid scars, including loss of E-cadherin expression and gain of mesenchymal markers vimentin and fibroblast-specific protein 1 in keloid epidermis [22]. Although we did not detect a significant difference in E-cadherin gene expression between normal and keloid keratinocytes, reduced levels of E-cadherin protein were observed in keloid epidermis. The difference in mRNA vs. protein expression levels is not surprising because cadherin switching, a prototypical feature of EMT, is regulated at both the transcriptional and post-transcriptional levels [19]. In the current study, the reductions observed in E-cadherin protein levels were variable among scars from different individuals; however, we did not observe any keloid scar tissue that completely lacked E-cadherin expression. This contrasts with the dramatic reduction in E-cadherin expression reported by Yan et al. [22]. The discrepant results may be due to differences in the techniques used for immunohistochemistry or due to differences in the patient populations used for each study.

Elevated expression of active β-catenin in keloid epidermis was previously reported by Chua et al. [76]. In the current study, we extend those results to show that active β-catenin expression in keloid epidermis is increased in epidermal cells in regions that exhibit reduced E-cadherin expression. E-cadherin is the main binding partner of β-catenin at the cell membrane. Together with α-catenin, this complex mediates cell-cell adhesion and links the actin cytoskeletons of neighboring cells. Reduction of E-cadherin levels disrupts cell adhesion and results in release of β-catenin from the membrane. Once dissociated from E-cadherin, β-catenin can accumulate in the cytoplasm and translocate to the nucleus to activate genes involved in EMT [77].

Expression of vimentin in epithelial cells is a hallmark of EMT, and the significantly elevated expression of *VIM* in keloid keratinocytes suggests induction of EMT in vitro. We observed elevated expression levels of vimentin in keloid scars and increased frequency of vimentin-positive epidermal cells, compared with normal skin. Yan et al. recently reported observing increased vimentin expression in keloid epidermis and in keloid microvessels [22]. Expression of vimentin in the vascular endothelium of keloid tissue was previously suggested as evidence of endothelial-to-mesenchymal transition, which, like EMT, has been proposed to contribute to fibrosis [78]. We observed localization of vimentin-positive cells in vessels of both keloid scars and normal skin. This is likely due to vimentin expression in endothelial cells, which has been previously reported [64], rather than the result of a pathological transition. In the current study, we observed numerous vimentin-positive cells localized to the BMZ in both keloid scars and normal skin in vivo, in association with apparent gaps in the basement membrane*.* Similar observations in hypertrophic scars were considered evidence of EMT-induced migration of cells across the BMZ [62]. However, in the current study, many of the vimentin-positive epidermal cells, particularly near the BMZ, were likely melanocytes or Langerhans cells. The results suggest that use of vimentin as a marker of EMT must be approached with caution, as it can be expressed in many different cell types [10]. The results also emphasize the importance of comparing keloid scar tissue with normal skin as a control, as certain phenotypes associated with EMT may also be present in some normal tissue samples.

The apparent increase of cells coexpressing vimentin and langerin in the suprabasal layers of keloid epidermis suggests that keloid scars may contain elevated numbers of Langerhans cells. Previous studies reported no difference in Langerhans cell density among keloids, non-keloid scars, and normal skin [79]. Although a large number of scars were included in that study, the ages of the patients and scar durations were not reported. In the current study, scars of varying duration were investigated, including active scars from relatively young patients, which may have influenced the levels of Langerhans cells. Confirmation of increased Langerhans cell density will require careful control of variables such as patient age, scar size, and scar duration in subsequent studies.

Although ultrastructural alterations of desmosomes and hemidesmosomes in keloid scars have been reported, we did not find evidence of significant disruptions of the epidermal-dermal BMZ in keloid scars. The apparent gaps in laminin-332 and integrin α6 basement membrane staining were likely due to the absence of detectable expression of these proteins in melanocytes and Langerhans cells in the BMZ. Discontinuities of collagen IV localization in the BMZ were not observed. EMT-associated basement membrane breakdown is considered an important step during tumor invasion and metastasis, enabling tumor cell detachment, migration, and dissemination to distant sites [60]. Although keloids are characterized by local spread beyond the original wound boundary, metastasis to distant sites does not occur. The lack of basement membrane breakdown in keloid scars may be a key factor that prevents metastatic spread of keloid scars.

## Conclusions

The observation that keloid scars exhibit many of the hallmarks of EMT, although failed to exhibit others, suggests a role for partial EMT in keloid scarring. A similar phenomenon was recently described in the epidermis of patients with systemic sclerosis, a fibrotic disease affecting the skin and other organs [80]. The simultaneous expression of both epithelial and mesenchymal traits has been referred to as a metastable state, which represents an intermediate stage of EMT [81, 82]. The metastable state is considered reversible upon removal of the EMT stimulus, compared with the “epigenetically fixed” state of complete EMT with transition to a mesenchymal phenotype [82]. The phenotype of activated keratinocytes during wound re-epithelialization has been considered a metastable state [83]. During wound healing, keratinocytes exhibit morphological changes, display reductions in numbers of cell-cell junctions, and become migratory, similar to cells undergoing EMT. In normal healing, this EMT-like metastable state is reversible; once re-epithelialization is complete, keratinocytes revert to a fully epithelial phenotype and re-establish stable cell-cell and cell-substrate contacts. The present study suggests that keloid keratinocytes are stalled in a metastable state, regulated by canonical and non-canonical TGF-β1 signaling pathways, which leads to pathological overhealing and localized fibroproliferation.

We do not yet know the signals upstream of TGF-β1 that initiate the EMT-like changes of keloid keratinocytes or fully understand the mechanisms that maintain their metastable phenotype. Excessive inflammation, which has long been thought to play a role in keloid pathology [84], is also implicated in EMT [85]. Infiltrating immune cells secrete cytokines that mediate EMT and support tumor cell invasion and migration [85]. Hypothetically, in genetically susceptible patients, excessive inflammation during wound healing may tip the balance of canonical and non-canonical TGF-β1 signaling, leading to an EMT-like state that, in the absence of inhibitory signals, leads to pathological overhealing. How the EMT-like state of keloid keratinocytes contributes to keloid pathology remains to be determined, but it has been previously shown that factors secreted by keloid keratinocytes can stimulate fibrotic responses in fibroblasts [86]. In keloid scars, TGF-β1-induced overexpression in keloid keratinocytes of secreted factors, such as *IL6* and *WNT5A*, may contribute to fibrosis directly, by stimulating signaling pathways in keloid fibroblasts that increase ECM production or indirectly by promoting an inflammatory microenvironment [87–89]. Current keloid treatments that inhibit the activity of fibroblasts may fail to provide long-term suppression of fibrosis if the EMT-like phenotype of keloid keratinocytes remains unchecked. Therapeutic interventions that target the EMT process to promote reversion to a stable epithelial phenotype in keloid keratinocytes may be required, in addition to treatments aimed at fibroblast activity, to fully suppress keloid scarring.

## Abbreviations

ADAM 19, a disintegrin and metallopeptidase domain 19 (ADAM19); ALK5, activin receptor-like kinase; ANOVA, analysis of variance; BMZ, basement membrane zone; CDH11, cadherin 11; EMT, epithelial-mesenchymal transition; ERK 1/2, extracellular signal-regulated kinase 1/2; FZD7, frizzled 7; HAS2, hyaluronan synthase 2; IL6, interleukin 6; MAPK, mitogen-activated protein kinase; qPCR, quantitative polymerase chain reaction; TGF-β1, transforming growth factor beta 1; VIM, vimentin; WNT5A, wingless-type MMTV integration site family, member 5A

## Additional files

Additional file 1: Additional methods. Additional file 2: Figure S1. Analysis of Smad3 phosphorylation in normal and keloid keratinocytes treated with TGF-β1 or SB525334, respectively. **A**. Western blot analysis of P-Smad3, Smad3, and β-actin (loading control). Representative samples are shown: lane 1, normal keratinocytes (untreated controls); lane 2, normal keratinocytes + 1.0 ng/ml TGF-β1; lane 3, keloid keratinocytes (untreated controls); lane 4, keloid keratinocytes + 1.0 μM SB525334. **B**. Quantitative analysis of Smad3 phosphorylation in treated cells vs. controls. Additional file 3: Figure S2. The gene encoding β-catenin is not differentially expressed between normal and keloid keratinocytes, but its expression is increased in keloid keratinocytes by TGF-β1. **A**. CTNNB1 expression in normal keratinocytes (NK; *N* = 8 strains) and keloid keratinocytes (KK; *N* = 8 strains) was determined by qPCR. No significant difference was observed. **B**. Treatment of keloid keratinocytes with TGF-β1 resulted in a significant increase in CTNNB1 expression. This increase was attenuated by treatment with U0126 to inhibit ERK1/2 signaling, but not by inhibition of p38 signaling with SB203580. Treatment with U0126 or SB203580 did not affect basal CTNNB1 expression levels. Statistically significant differences are indicated by symbols: **p* < 0.001; ^#^ *p* < 0.05.

## References

[1] Butler PD, Longaker MT, Yang GP (2008). Current progress in keloid research and treatment. J Am Coll Surg.

[2] Gauglitz GG, Korting HC, Pavicic T, Ruzicka T, Jeschke MG (2011). Hypertrophic scarring and keloids: pathomechanisms and current and emerging treatment strategies. Mol Med.

[3] Stella M, Castagnoli C, Gangemi EN (2008). Postburn scars: an update. Int J Low Extrem Wounds.

[4] Davis SA, Feldman SR, McMichael AJ (2013). Management of keloids in the United States, 1990–2009: an analysis of the National Ambulatory Medical Care Survey. Dermatol Surg.

[5] Slemp AE, Kirschner RE (2006). Keloids and scars: a review of keloids and scars, their pathogenesis, risk factors, and management. Curr Opin Pediatr.

[6] Chike-Obi CJ, Cole PD, Brissett AE (2009). Keloids: pathogenesis, clinical features, and management. Semin Plast Surg.

[7] Perez AD, Hirschman C (2009). The changing racial and ethnic composition of the US population: emerging American identities. Popul Dev Rev.

[8] Durani P, Bayat A (2008). Levels of evidence for the treatment of keloid disease. J Plast Reconstr Aesthet Surg.

[9] Hahn JM, Glaser K, McFarland KL, Aronow BJ, Boyce ST, Supp DM (2013). Keloid-derived keratinocytes exhibit an abnormal gene expression profile consistent with a distinct causal role in keloid pathology. Wound Rep Reg.

[10] Zeisberg M, Neilson EG (2009). Biomarkers for epithelial-mesenchymal transitions. J Clin Invest.

[11] Kalluri R, Weinberg RA (2009). The basics of epithelial-mesenchymal transition. J Clin Invest.

[12] Wendt MK, Tian M, Schiemann WP (2012). Deconstructing the mechanisms and consequences of TGF-beta-induced EMT during cancer progression. Cell Tissue Res.

[13] Morrison CD, Parvani JG, Schiemann WP (2013). The relevance of the TGF-beta Paradox to EMT-MET programs. Cancer Lett.

[14] Wynn TA (2008). Cellular and molecular mechanisms of fibrosis. J Pathol.

[15] Burns WC, Thomas MC (2010). The molecular mediators of type 2 epithelial to mesenchymal transition (EMT) and their role in renal pathophysiology. Expert Rev Mol Med..

[16] Guarino M, Tosoni A, Nebuloni M (2009). Direct contribution of epithelium to organ fibrosis: epithelial-mesenchymal transition. Hum Pathol.

[17] Chapman HA (2011). Epithelial-mesenchymal interactions in pulmonary fibrosis. Annu Rev Physiol.

[18] Thiery JP, Acloque H, Huang RY, Nieto MA (2009). Epithelial-mesenchymal transitions in development and disease. Cell.

[19] Wheelock MJ, Shintani Y, Maeda M, Fukumoto Y, Johnson KR (2008). Cadherin switching. J Cell Sci.

[20] Groger CJ, Grubinger M, Waldhor T, Vierlinger K, Mikulits W (2012). Meta-analysis of gene expression signatures defining the epithelial to mesenchymal transition during cancer progression. PLoS One.

[21] Hellstrom M, Hellstrom S, Engstrom-Laurent A, Bertheim U (2014). The structure of the basement membrane zone differs between keloids, hypertrophic scars and normal skin: a possible background to an impaired function. J Plast Reconstr Aesthet Surg.

[22] Yan L, Cao R, Wang L, Liu Y, Pan B, Yin Y (2015). Epithelial-mesenchymal transition (EMT) in keloid tissues and TGF-beta1-induced hair follicle outer root sheath keratinocytes. Wound Repair Regen.

[23] Xu J, Lamouille S, Derynck R (2009). TGF-beta-induced epithelial to mesenchymal transition. Cell Res.

[24] Wendt MK, Allington TM, Schiemann WP (2009). Mechanisms of the epithelial-mesenchymal transition by TGF-beta. Future Oncol.

[25] Bran GM, Goessler UR, Hormann K, Riedel F, Sadick H (2009). Keloids: current concepts of pathogenesis (review). Int J Mol Med.

[26] Lee TY, Chin GS, Kim WJ, Chau D, Gittes GK, Longaker MT (1999). Expression of transforming growth factor beta 1, 2, and 3 proteins in keloids. Ann Plast Surg.

[27] Babu M, Diegelmann R, Oliver N (1992). Keloid fibroblasts exhibit an altered response to TGF-beta. J Invest Dermatol.

[28] He S, Liu X, Yang Y, Huang W, Xu S, Yang S (2010). Mechanisms of transforming growth factor beta(1)/Smad signalling mediated by mitogen-activated protein kinase pathways in keloid fibroblasts. Br J Dermatol.

[29] Chin GS, Liu W, Peled Z, Lee TY, Steinbrech DS, Hsu M (2001). Differential expression of transforming growth factor-beta receptors I and II and activation of Smad 3 in keloid fibroblasts. Plast Reconstr Surg.

[30] Daian T, Ohtsuru A, Rogounovitch T, Ishihara H, Hirano A, Kiyama-Uchida Y (2003). Insulin-like growth factor-I enhances transforming growth factor-beta-induced extracellular matrix protein production through the P38/activating transcription factor-2 signaling pathway in keloid fibroblasts. J Invest Dermatol.

[31] Fukawa T, Kajiya H, Ozeki S, Ikebe T, Okabe K (2012). Reactive oxygen species stimulates epithelial mesenchymal transition in normal human epidermal keratinocytes via TGF-beta secretion. Exp Cell Res.

[32] Porsch H, Bernert B, Mehic M, Theocharis AD, Heldin CH, Heldin P (2013). Efficient TGFbeta-induced epithelial-mesenchymal transition depends on hyaluronan synthase HAS2. Oncogene.

[33] Guo N, Kanter D, Funderburgh ML, Mann MM, Du Y, Funderburgh JL (2007). A rapid transient increase in hyaluronan synthase-2 mRNA initiates secretion of hyaluronan by corneal keratocytes in response to transforming growth factor beta. J Biol Chem.

[34] Supp DM, Hahn JM, McFarland KL, Glaser K (2014). Inhibition of hyaluronan synthase 2 reduces the abnormal migration rate of keloid keratinocytes. J Burn Care Res.

[35] Zavadil J, Bitzer M, Liang D, Yang YC, Massimi A, Kneitz S (2001). Genetic programs of epithelial cell plasticity directed by transforming growth factor-beta. Proc Natl Acad Sci U S A.

[36] Boyce ST (1999). Methods for the serum-free culture of keratinocytes and transplantation of collagen-GAG-based skin substitutes. Methods Mol Med.

[37] Hoq MI, Niyonsaba F, Ushio H, Aung G, Okumura K, Ogawa H (2011). Human catestatin enhances migration and proliferation of normal human epidermal keratinocytes. J Dermatol Sci.

[38] Livak KJ, Schmittgen TD (2001). Analysis of relative gene expression data using real-time quantitative PCR and the 2(−delta delta C(T)) method. Methods.

[39] Van NM, Meeldijk J, van der ZR, Destree O, Clevers H (2002). Wnt signaling controls the phosphorylation status of beta-catenin. J Biol Chem.

[40] RL OD, McCormick A, Mukhopadhyay A, Woodhouse LC, Moat M, Grundy A (2014). The use of ovarian cancer cells from patients undergoing surgery to generate primary cultures capable of undergoing functional analysis. PLoS One.

[41] Lau SK, Chu PG, Weiss LM (2008). Immunohistochemical expression of Langerin in Langerhans cell histiocytosis and non-Langerhans cell histiocytic disorders. Am J Surg Pathol.

[42] Bouchard B, Fuller BB, Vijayasaradhi S, Houghton AN (1989). Induction of pigmentation in mouse fibroblasts by expression of human tyrosinase cDNA. J Exp Med.

[43] Goldfinger LE, Stack MS, Jones JC (1998). Processing of laminin-5 and its functional consequences: role of plasmin and tissue-type plasminogen activator. J Cell Biol.

[44] Aumailley M, Timpl R, Sonnenberg A (1990). Antibody to integrin alpha 6 subunit specifically inhibits cell-binding to laminin fragment 8. Exp Cell Res.

[45] Zoltan-Jones A, Huang L, Ghatak S, Toole BP (2003). Elevated hyaluronan production induces mesenchymal and transformed properties in epithelial cells. J Biol Chem.

[46] Li L, Qi L, Liang Z, Song W, Liu Y, Wang Y (2015). Transforming growth factor-beta1 induces EMT by the transactivation of epidermal growth factor signaling through HA/CD44 in lung and breast cancer cells. Int J Mol Med.

[47] Moustakas A, Heldin P (2014). TGFbeta and matrix-regulated epithelial to mesenchymal transition. Biochim Biophys Acta.

[48] Schneider DJ, Wu M, Le TT, Cho SH, Brenner MB, Blackburn MR (2012). Cadherin-11 contributes to pulmonary fibrosis: potential role in TGF-beta production and epithelial to mesenchymal transition. FASEB J.

[49] Ford CE, Punnia-Moorthy G, Henry CE, Llamosas E, Nixdorf S, Olivier J (2014). The non-canonical Wnt ligand, Wnt5a, is upregulated and associated with epithelial to mesenchymal transition in epithelial ovarian cancer. Gynecol Oncol.

[50] Deng B, Zhang S, Miao Y, Zhang Y, Wen F, Guo K (2015). Down-regulation of Frizzled-7 expression inhibits migration, invasion, and epithelial-mesenchymal transition of cervical cancer cell lines. Med Oncol.

[51] Schiffmacher AT, Padmanabhan R, Jhingory S, Taneyhill LA (2014). Cadherin-6B is proteolytically processed during epithelial-to-mesenchymal transitions of the cranial neural crest. Mol Biol Cell.

[52] Kurohara K, Komatsu K, Kurisaki T, Masuda A, Irie N, Asano M (2004). Essential roles of Meltrin beta (ADAM19) in heart development. Dev Biol.

[53] Ramdas V, McBride M, Denby L, Baker AH (2013). Canonical transforming growth factor-beta signaling regulates disintegrin metalloprotease expression in experimental renal fibrosis via miR-29. Am J Pathol.

[54] Jung HY, Fattet L, Yang J (2015). Molecular pathways: linking tumor microenvironment to epithelial-mesenchymal transition in metastasis. Clin Cancer Res.

[55] Yadav A, Kumar B, Datta J, Teknos TN, Kumar P (2011). IL-6 promotes head and neck tumor metastasis by inducing epithelial-mesenchymal transition via the JAK-STAT3-SNAIL signaling pathway. Mol Cancer Res.

[56] Grygielko ET, Martin WM, Tweed C, Thornton P, Harling J, Brooks DP (2005). Inhibition of gene markers of fibrosis with a novel inhibitor of transforming growth factor-beta type I receptor kinase in puromycin-induced nephritis. J Pharmacol Exp Ther.

[57] O'Kane D, Jackson MV, Kissenpfennig A, Spence S, Damkat-Thomas L, Tolland JP (2014). SMAD inhibition attenuates epithelial to mesenchymal transition by primary keratinocytes in vitro. Exp Dermatol.

[58] Staal FJ, Noort MM, Strous GJ, Clevers HC (2002). Wnt signals are transmitted through N-terminally dephosphorylated beta-catenin. EMBO Rep.

[59] Nakaya Y, Sukowati EW, Wu Y, Sheng G (2008). RhoA and microtubule dynamics control cell-basement membrane interaction in EMT during gastrulation. Nat Cell Biol.

[60] Spaderna S, Schmalhofer O, Hlubek F, Berx G, Eger A, Merkel S (2006). A transient, EMT-linked loss of basement membranes indicates metastasis and poor survival in colorectal cancer. Gastroenterology.

[61] Rowe RG, Weiss SJ (2008). Breaching the basement membrane: who, when and how?. Trends Cell Biol.

[62] Yan C, Grimm WA, Garner WL, Qin L, Travis T, Tan N (2010). Epithelial to mesenchymal transition in human skin wound healing is induced by tumor necrosis factor-alpha through bone morphogenic protein-2. Am J Pathol.

[63] Sugawara K, Tsuruta D, Ishii M, Jones JC, Kobayashi H (2008). Laminin-332 and -511 in skin. Exp Dermatol.

[64] Terpe HJ, Stark H, Ruiz P, Imhof BA (1994). Alpha 6 integrin distribution in human embryonic and adult tissues. Histochemistry.

[65] Plettenberg A, Ballaun C, Pammer J, Mildner M, Strunk D, Weninger W (1995). Human melanocytes and melanoma cells constitutively express the Bcl-2 proto-oncogene in situ and in cell culture. Am J Pathol.

[66] Juckett G, Hartman-Adams H (2009). Management of keloids and hypertrophic scars. Am Fam Physician.

[67] Niessen FB, Andriessen MP, Schalkwijk J, Visser L, Timens W (2001). Keratinocyte-derived growth factors play a role in the formation of hypertrophic scars. J Pathol.

[68] Ghahary A, Ghaffari A (2007). Role of keratinocyte-fibroblast cross-talk in development of hypertrophic scar. Wound Repair Regen.

[69] Mustoe TA, Gurjala A (2011). The role of the epidermis and the mechanism of action of occlusive dressings in scarring. Wound Repair Regen.

[70] Meyer M, Muller AK, Yang J, Sulcova J, Werner S (2011). The role of chronic inflammation in cutaneous fibrosis: fibroblast growth factor receptor deficiency in keratinocytes as an example. J Investig Dermatol Symp Proc.

[71] Kim S, Lee J, Jeon M, Nam SJ, Lee JE (2015). Elevated TGF-beta1 and -beta2 expression accelerates the epithelial to mesenchymal transition in triple-negative breast cancer cells. Cytokine.

[72] Serra R, Easter SL, Jiang W, Baxley SE (2011). Wnt5a as an effector of TGFbeta in mammary development and cancer. J Mammary Gland Biol Neoplasia.

[73] Yamada D, Kobayashi S, Wada H, Kawamoto K, Marubashi S, Eguchi H (2013). Role of crosstalk between interleukin-6 and transforming growth factor-beta 1 in epithelial-mesenchymal transition and chemoresistance in biliary tract cancer. Eur J Cancer.

[74] Chan MW, Huang YW, Hartman-Frey C, Kuo CT, Deatherage D, Qin H (2008). Aberrant transforming growth factor beta1 signaling and SMAD4 nuclear translocation confer epigenetic repression of ADAM19 in ovarian cancer. Neoplasia.

[75] Tsukada S, Westwick JK, Ikejima K, Sato N, Rippe RA (2005). SMAD and p38 MAPK signaling pathways independently regulate alpha1(I) collagen gene expression in unstimulated and transforming growth factor-beta-stimulated hepatic stellate cells. J Biol Chem.

[76] Chua AW, Ma D, Gan SU, Fu Z, Han HC, Song C (2011). The role of R-spondin2 in keratinocyte proliferation and epidermal thickening in keloid scarring. J Invest Dermatol.

[77] Scanlon CS, Van Tubergen EA, Inglehart RC, D'Silva NJ (2013). Biomarkers of epithelial-mesenchymal transition in squamous cell carcinoma. J Dent Res.

[78] Lee WJ, Park JH, Shin JU, Noh H, Lew DH, Yang WI (2015). Endothelial-to-mesenchymal transition induced by Wnt 3a in keloid pathogenesis. Wound Repair Regen.

[79] Bagabir R, Byers RJ, Chaudhry IH, Muller W, Paus R, Bayat A (2012). Site-specific immunophenotyping of keloid disease demonstrates immune upregulation and the presence of lymphoid aggregates. Br J Dermatol.

[80] Nikitorowicz-Buniak J, Denton CP, Abraham D, Stratton R (2015). Partially evoked epithelial-mesenchymal transition (EMT) is associated with increased TGFbeta signaling within lesional scleroderma skin. PLoS One.

[81] Jordan NV, Johnson GL, Abell AN (2011). Tracking the intermediate stages of epithelial-mesenchymal transition in epithelial stem cells and cancer. Cell Cycle.

[82] Thomson S, Petti F, Sujka-Kwok I, Mercado P, Bean J, Monaghan M (2011). A systems view of epithelial-mesenchymal transition signaling states. Clin Exp Metastasis.

[83] Savagner P (2015). Epithelial-mesenchymal transitions: from cell plasticity to concept elasticity. Curr Top Dev Biol.

[84] Shih B, Garside E, McGrouther DA, Bayat A (2010). Molecular dissection of abnormal wound healing processes resulting in keloid disease. Wound Repair Regen.

[85] Lopez-Novoa JM, Nieto MA (2009). Inflammation and EMT: an alliance towards organ fibrosis and cancer progression. EMBO Mol Med.

[86] Lim IJ, Phan TT, Bay BH, Qi R, Huynh H, Tan WT (2002). Fibroblasts cocultured with keloid keratinocytes: normal fibroblasts secrete collagen in a keloidlike manner. Am J Physiol Cell Physiol.

[87] Duncan MR, Berman B (1991). Stimulation of collagen and glycosaminoglycan production in cultured human adult dermal fibroblasts by recombinant human interleukin 6. J Invest Dermatol.

[88] Igota S, Tosa M, Murakami M, Egawa S, Shimizu H, Hyakusoku H (2013). Identification and characterization of Wnt signaling pathway in keloid pathogenesis. Int J Med Sci.

[89] Ghazizadeh M, Tosa M, Shimizu H, Hyakusoku H, Kawanami O (2007). Functional implications of the IL-6 signaling pathway in keloid pathogenesis. J Invest Dermatol.

