# Supplementary material for: Partial epithelial-mesenchymal transition in keloid scars: regulation of keloid keratinocyte gene expression by transforming growth factor-β1
Source: Burns Trauma. 2016 Aug 23;4(1):30. doi: 10.1186/s41038-016-0055-7 (PMC4994224; doi:10.1186/s41038-016-0055-7)
Supplement: Additional file 1: — Additional methods. [file 41038_2016_55_MOESM1_ESM.docx]

**Additional Files**

**Additional Methods**

Western blot analysis was performed to analyze phosphorylation of Smad3 in normal and keloid keratinocytes treated with 1.0 ng/ml TGF-β1 or 1 mM SB525334, respectively. Western blot analysis utilized the Pierce Fast Western Blot Kit, SuperSignal West Dura, Rabbit (Thermo Scientific), with Anti-Smad3 Rabbit Monoclonal antibody (Abcam, Cambridge, MA; catalog #ab40854), Anti-Smad3 (phospho S423 + S425) antibody (Abcam; catalog #ab52903), and Anti-β-Actin (Abcam; catalog #ab8227). Blots were stripped between antibodies using Restore PLUS Western Blot Stripping Buffer (Thermo Scientific). Each band was quantified using Image J (https://imagej.nih.gov/ij/index.html), and values for Smad3 and P-Smad3 were normalized to the β-actin values. The ratios of normalized values for P-Smad/Smad were plotted.
