# Supplementary figures and images for: Partial epithelial-mesenchymal transition in keloid scars: regulation of keloid keratinocyte gene expression by transforming growth factor-β1
Source: Burns Trauma. 2016 Aug 23;4(1):30. doi: 10.1186/s41038-016-0055-7 (PMC4994224; doi:10.1186/s41038-016-0055-7)

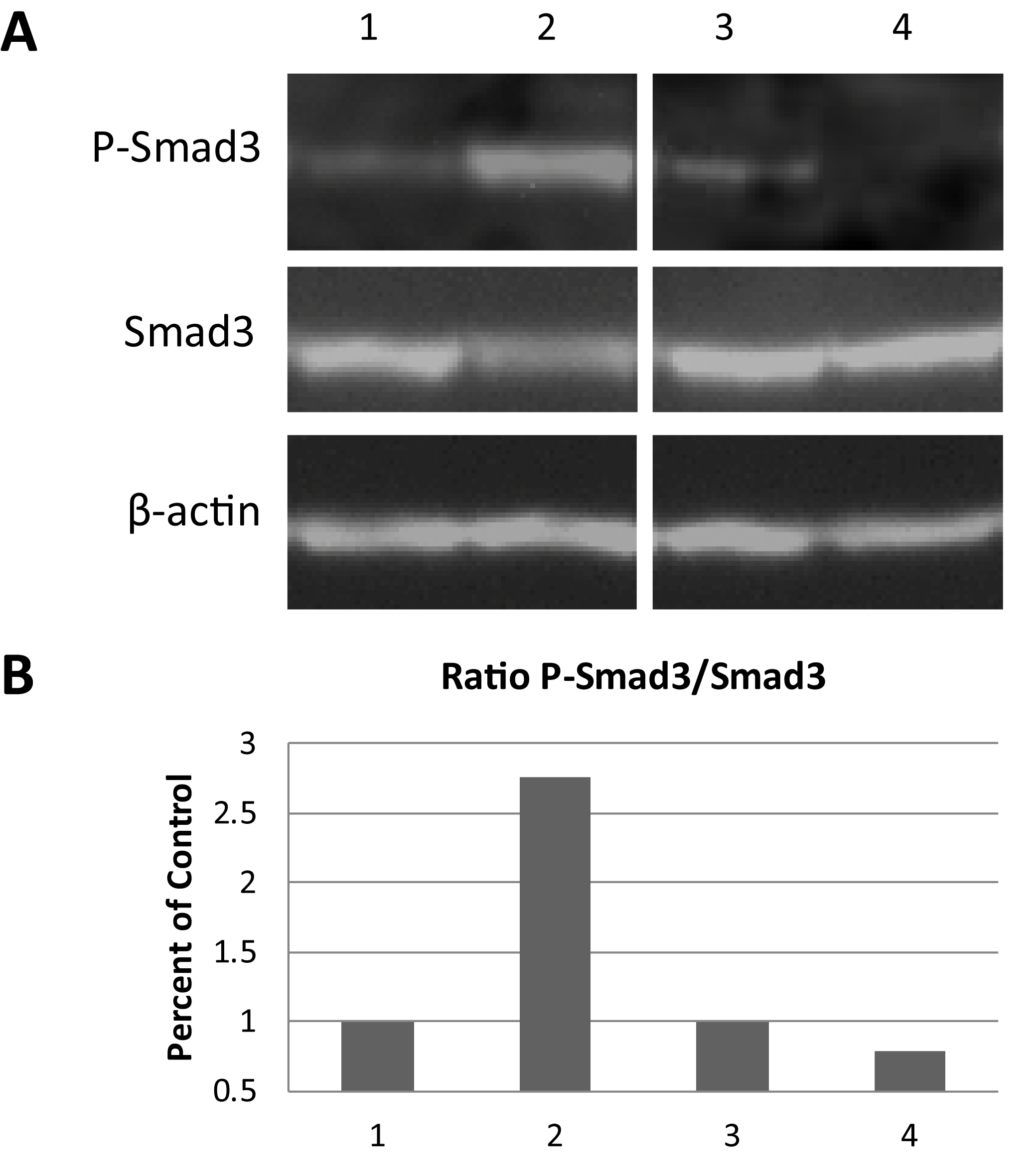

Supplement: Additional file 2: Figure S1. — Analysis of Smad3 phosphorylation in normal and keloid keratinocytes treated with TGF-β1 or SB525334, respectively. A. Western blot analysis of P-Smad3, Smad3, and β-actin (loading control). Representative samples are shown: lane 1, normal keratinocytes (untreated controls); lane 2, normal keratinocytes + 1.0 ng/ml TGF-β1; lane 3, keloid keratinocytes (untreated controls); lane 4, keloid keratinocytes + 1.0 μM SB525334. B. Quantitative analysis of Smad3 phosphorylation in treated cells vs. controls. [file 41038_2016_55_MOESM2_ESM.tif]

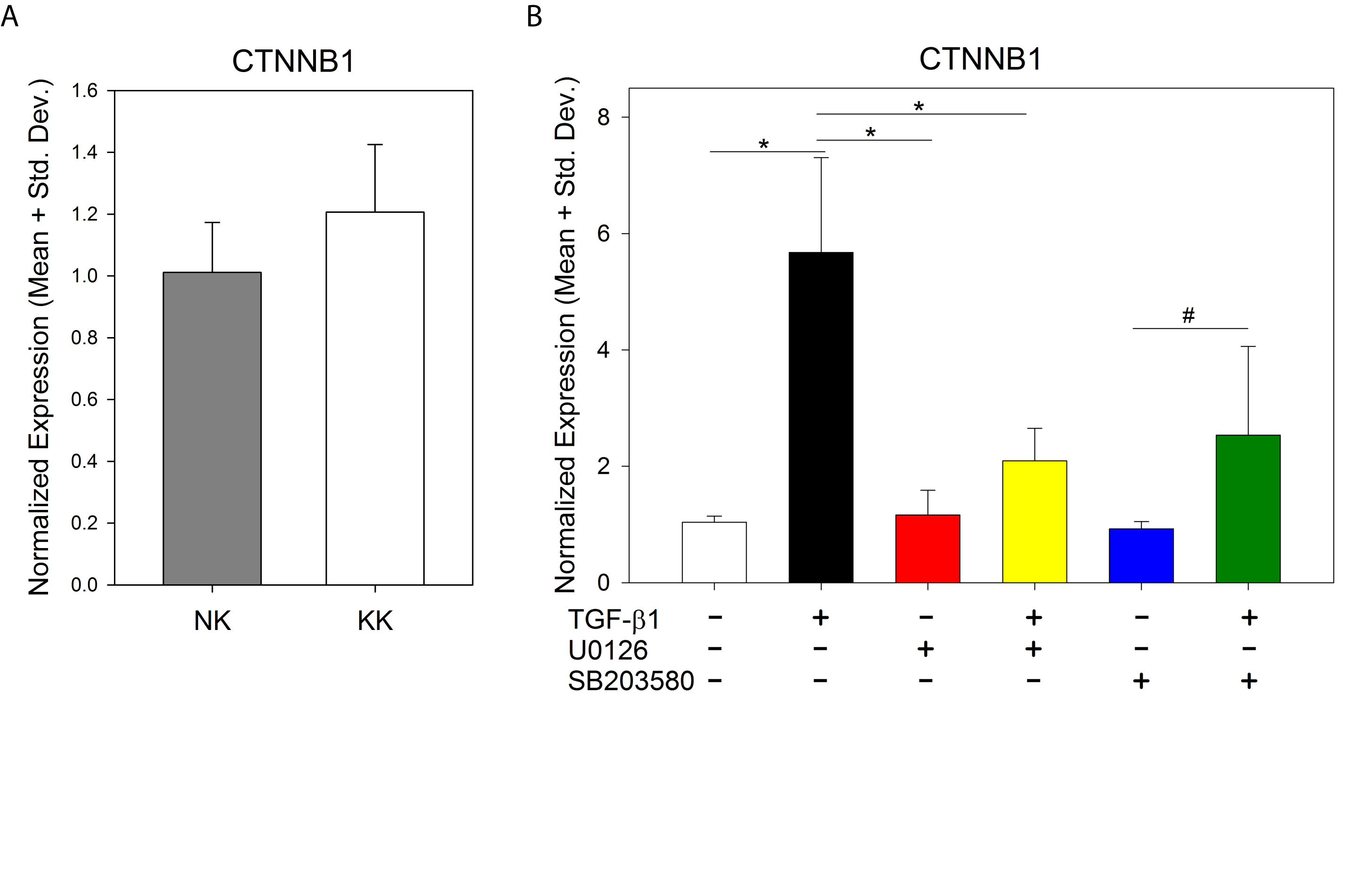

Supplement: Additional file 3: Figure S2. — The gene encoding β-catenin is not differentially expressed between normal and keloid keratinocytes, but its expression is increased in keloid keratinocytes by TGF-β1. A. CTNNB1 expression in normal keratinocytes (NK; N = 8 strains) and keloid keratinocytes (KK; N = 8 strains) was determined by qPCR. No significant difference was observed. B. Treatment of keloid keratinocytes with TGF-β1 resulted in a significant increase in CTNNB1 expression. This increase was attenuated by treatment with U0126 to inhibit ERK1/2 signaling, but not by inhibition of p38 signaling with SB203580. Treatment with U0126 or SB203580 did not affect basal CTNNB1 expression levels. Statistically significant differences are indicated by symbols: *p < 0.001; # p < 0.05. [file 41038_2016_55_MOESM3_ESM.tif]
